# Navigating Cardiovascular Challenges of Obesity: Exploring Preventive Approaches

**DOI:** 10.2174/0118715303317750250210055338

**Published:** 2025-03-19

**Authors:** Vibha Sinha, Shubhojeet Roy, Sapnita Shinde, Deepankar Mondal, Vineeta Dixit, Deepak Dwivedi, Sanjay Kumar Pandey, Rakesh Kumar Gupta, Naveen Kumar Vishwakarma, Dhananjay Shukla

**Affiliations:** 1 Department of Biotechnology, Guru Ghasidas Vishwavidyalaya Bilaspur, C.G., 4965001, India;; 2 Department of Biochemistry and Molecular Biology, University of California, Riverside, 92507, United States;; 3 Department of Botany, Nilamber Pitamber University, Maidininagar, Jharkhand, 822114, India;; 4 Department of Paediatrics, Shyam Shah Medical College, Rewa, M.P., 486001, India;; 5 Department of Multidisplinary Research Unit, Shyam Shah Medical College, Rewa, M.P.,486001, India;; 6 Department of Pathology and Lab Medicines, All India Institute of Medical Sciences Raipur AIIMS, Raipur, CG, 492009, India

**Keywords:** Cardiovascular illnesses, obesity, metabolic diseases, coronary artery disease, heart failure, hypertension, stroke, COVID-19, lifestyle management

## Abstract

The global prevalence of obesity has surged to epidemic proportions, posing a significant threat to public health in the twenty-first century. Beyond its established association with metabolic diseases, obesity profoundly impacts cardiovascular health, serving as a major risk factor for various cardiovascular illnesses (CVDs), including coronary artery disease, heart failure, hypertension, and stroke. Mechanistically, obesity triggers a cascade of pathophysiological processes, including chronic inflammation and insulin resistance, exacerbating atherosclerosis and endothelial dysfunction. Moreover, obesity correlates with metabolic abnormalities that further elevate the risk of cardiovascular events. As global community has faced the COVID-19 pandemic, and thus, the aftereffects of the pandemic might pose a spectrum of post-viral complications, including cardiovascular sequelae such as myocarditis and arrhythmias. Considering the intersectionality of obesity, COVID-19, and cardiovascular health are imperative, particularly as obese individuals face heightened risks of severe post-COVID-19 effects and subsequent cardiovascular complications. Lifestyle management emerges as a cornerstone in preventing and managing obesity-related cardiovascular risks, encompassing dietary modifications, physical activity, behavioural therapies, and patient education. Embracing innovative approaches, including modulation of gut microbiota and novel drug developments, holds promise in addressing the intricate nexus between obesity and cardiovascular diseases. This review underscores the paramount importance of lifestyle interventions over pharmacological measures, advocating for a comprehensive approach involving healthcare practitioners, researchers, and policymakers to mitigate the long-term cardiovascular consequences of obesity and COVID-19.

## 1 INTRODUCTION

Obesity has reached an epidemic level around the world, creating a significant risk to world health in the twenty-first century. Obesity's varied repercussions go far beyond its well-documented relationship with metabolic diseases and reduced quality of life. One of the most important issues is the devastating effect of obesity on cardiovascular health. Obesity, as described by the World Obesity Foundation, is a buildup of excess body weight which is mostly due to the accumulation of fat. In contrast, the World Health Organisation (WHO) and the Centre for Disease Control (CDC) roughly define obese individuals as someone having a BMI (body mass index) of 30 or more and overweight individuals having a BMI of 25 or more. Obesity is recognized as a major risk factor for a variety of cardiovascular illnesses (CVDs), including coronary artery disease, heart failure, hypertension, and stroke. The underlying mechanisms that relate obesity to CVDs are complex and involve a wide range of pathophysiological processes. Adipose tissue, formerly thought to be only a passive energy storage depot, is now recognized as an active endocrine organ, generating a slew of pro-inflammatory cytokines and adipokines that promote chronic low- grade inflammation and insulin resistance [1, 2]. This inflammatory environment encourages the progression of atherosclerosis and endothelial dysfunction, both of which are critical events in the pathophysiology of CVDs.

Furthermore, obesity is linked to a variety of metabolic abnormalities, including dyslipidemia, hyperinsulinemia, and dysregulated glucose metabolism, all of which raise the risk of atherosclerotic plaque development and thrombotic events. Furthermore, obesity-related comorbidities such as sleep apnea, non-alcoholic fatty liver disease, and chronic kidney disease worsen the cardiovascular risk picture. The worrisome rise in obesity rates needs a proactive strategy to mitigate its negative cardiovascular implications [3, 4].

Apart from that, in the post-COVID-19 global health scenario, there has been increasing evidence suggesting that individuals recovered or recovering from the virus may experience lingering health issues, commonly known as post-COVID complications. These complications are diverse and can affect various organs, including the heart. Cardiovascular complications, such as myocarditis, arrhythmias, and blood clotting disorders, have been observed in some COVID-19 survivors. These complications can exacerbate existing cardiovascular conditions or create new challenges for individuals previously unaffected by heart problems. Therefore, it is crucial to consider the intersectionality of obesity, cardiovascular health, and post-COVID complications [5, 6]. Obese individuals, being at a higher risk for severe COVID-19 outcomes, might face heightened risks of developing cardiovascular complications during and after their recovery from the virus. Understanding the interplay between obesity, COVID-19, and cardiac health is essential for healthcare practitioners, researchers, and policymakers to develop comprehensive strategies aimed at mitigating the long-term cardiovascular consequences of both obesity and COVID-19.

Emphasizing lifestyle management holds paramount importance in preventing various health conditions. Dietary modifications and physical activity serve as foundational elements in obesity therapy, offering significant potential to improve cardiovascular outcomes. Additionally, behavioral therapies and patient education play a crucial role in nurturing a long-term commitment to healthy behaviors. For individuals dealing with significant obesity or uncontrolled risk factors, prioritizing lifestyle changes should take precedence over considering pharmacological therapies or surgical procedures. Recent research efforts have delved into innovative areas such as regulating gut microbiota and developing new drugs to address the intricate connections between obesity and cardiovascular diseases. Recognizing the underlying processes and identifying high-risk populations are pivotal in designing effective preventive methods to enhance cardiovascular health amidst the obesity pandemic. In summary, this review underscores the vital role of lifestyle management in disease prevention, highlighting the need to focus on lifestyle changes over pharmacological interventions. A comprehensive approach involving healthcare practitioners, academics, and policymakers is essential to effectively tackle this growing public health concern [7-9].

## 2 GLOBAL TRENDS IN OBESITY AND CARDIOVASCULAR DISEASE

Obesity prevalence has escalated critically worldwide and has been identified as an important health issue that needs better treatment and prevention strategies. The trend of obesity has been steadily increasing over the past few decades, and it is now considered a global epidemic. In both developed and developing countries, there has been a significant increase in the prevalence of obesity, particularly in urban areas [10]. In accordance with the World Obesity Federation's World Obesity Atlas 2023, if prevention and treatment approaches do not improve, the global economic burden of overweight and obesity will reach $4.32 trillion annually by 2035. This is similar to the impact of COVID-19 in 2020, which is estimated to be around 3% of the global GDP. If present trends continue, the majority of the world's population (51%, or more than 4 billion people) will be overweight or obese by 2035. Obesity will affect one in every four persons (almost two billion). In China, one research of 12,543 participants followed for 22 years found that the prevalence of age-adjusted obesity increased from 2.15% to 13.99% in both sexes, rising from 2.78 to 13.22% in females and 1.46 to 14.99% in males. Since 2000, the overweight rate of African children under the age of five has increased by 24%. In 2019, nearly half of Asian children under the age of five were obese or overweight. According to WHO data from Sub-Saharan Africa, the prevalence of overweight and obesity in adults and stunting, underweight, and wasting in children are inversely related. Despite the efforts by multiple organizations at the global level, the prevalence of obesity continues to increase incessantly [11]. India ranks 3^rd^ in the Global Obesity Index. It is estimated that approximately 135 million Indians are affected by obesity, which differs due to age, gender, geographic environment, genetics, socioeconomic status, lifestyle, *etc*. As per the 2015 ICMR-INDIAN report, central obesity and its prevalence rate varied from 16.9-36.3% and 11.8-31.3% respectively. Studies have indicated that women are at higher risk of being affected by obesity. Obesity has thus been identified as a medical and financial burden. The World Health Organisation (WHO) estimates that roughly 1.9 billion adults globally (nearly 39% of the world's population) are overweight. More than 650 million of these people (about 13% of the global population) are obese [12, 13]. Abdominal obesity significantly contributes to CVD.

Obesity prevalence trends in the United States and across the globe demonstrate the tremendous influence that obesity will continue to have on cardiovascular incidence and prevalence worldwide. CVD is expected to cause 17.9 million deaths per year. The Global Burden of Disease study estimate of an age-standardized CVD death rate of 272 per 100,000 in India is higher than the global average of 235 per 100,000. The primary cause of disquiet in CVD is its onset at an early age, progression at high speed, and elevated fatality rate. A study comparatively analysing the statistics of 1990 against 2016 in the context of total death and total disability-adjusted life years (DALYs) reported an elevation of 15.2% *v/s* 28.1% and 6.9% *v/s* 14.1%, respectively. However, with adequate physical activity, proper diet, and awareness, it could be preventable. Desperate efforts are needed to develop a comprehensive understanding of obesity emerging in endemic proportions to manage it [14-16] effectively.

## 3 RISK FACTORS FOR OBESITY AND CARDIOVASCULAR DISEASE

### 3.1 Diet

Diet is an important factor responsible for influencing health in various ways and it extends its impact beyond mere sustenance. The food choices we make create a profound effect on our mental and physical well-being. In recent years, the cases of obesity have increased significantly, which made it a focal point of research and public health campaigns, as understanding the connection between diet and healthier well-being could adverse the effects of obesity. A balanced diet ensures essential nutrition for growth, development, and maintenance. A healthy or balanced diet contains composites of bioactive compounds, including starches, vitamins, minerals, phytochemicals, and antioxidants. This cocktail of components in a balanced diet is vital for supporting bodily functions and strengthening the immune system. Whilst a balanced diet also responds inversely to obesity, type 2 diabetes, and cardiovascular diseases [17]. An imbalanced diet identified by increased levels of added sugars, saturated and trans fatty acids, and a lack of essential nutrients can lead to excessive calorie intake and energy density. Over time, consistent consumption of such diets can result in significant weight gain and trigger chronic inflammation, increasing the risk of cardiovascular diseases. Among the various unbalanced diets contributing to substantial weight gain, the Western diet stands out as a prominent culprit. This diet is notably high in sodium and unhealthy fats, and ultra-processed foods while being deficient in fruits and vegetables.

Additionally, it is rich in calorie-dense foods and added sugars [18, 19]. While fruits and vegetables contain natural sugars, they also offer vital nutrients like vitamins, minerals, and antioxidants. In contrast, added sugars only contribute to surplus sugar intake without any nutritional benefits. The Western diet was shown to have a higher proportion of saturated and trans fats. These fats not only add excessive calories to the diet but also elevate levels of low-density lipoprotein, thereby promoting the development of atherosclerosis. According to emerging epidemiological data, a higher intake of ultra-processed, saturated, and trans-fat foods is related to an elevated risk of cardiovascular disease [CVD]. According to the Framingham Offspring Study and the French NutriNet-Santé cohort study, each additional daily serving of ultra-processed meals is related to a 7% and 12% increase in CVD risk, respectively. Furthermore, meta-analyses have shown that specific ultra-processed goods, such as processed meat and sugar-sweetened beverages, as well as components prevalent in ultra-processed foods, such as trans fats and sodium, can increase the risk of CVD. Furthermore, epidemiologic research has discovered links between ultra-processed meals and various CVD risk factors such as obesity, hypertension, and metabolic syndrome [20-23].

Dietary patterns and food processing have an impact on the nutritional content, physical structure, and chemical composition of foods, which might have an impact on their health consequences. Changes in processed foods may also have an impact on long-term dietary habits, satiety signals, and reward systems involved with food consumption. The link between ultra-processed meals, high levels of trans fats, sugar, and cardiovascular health is complicated and complex, with several substances and properties of these foods interacting in ways that are not fully understood. Atherogenesis and the evolution of cardiovascular disease (CVD) are multifaceted processes involving numerous physiological pathways. Several factors coexist and interact to worsen each other, including metabolic, proinflammatory, prothrombotic, pro-oxidative, and endothelial dysfunction. There are multiple complexities at work; for example, differing levels of glucose metabolism changes can activate diverse inflammatory patterns, and immunological factors interact bidirectionally with gut flora.

**Fig. (1):**
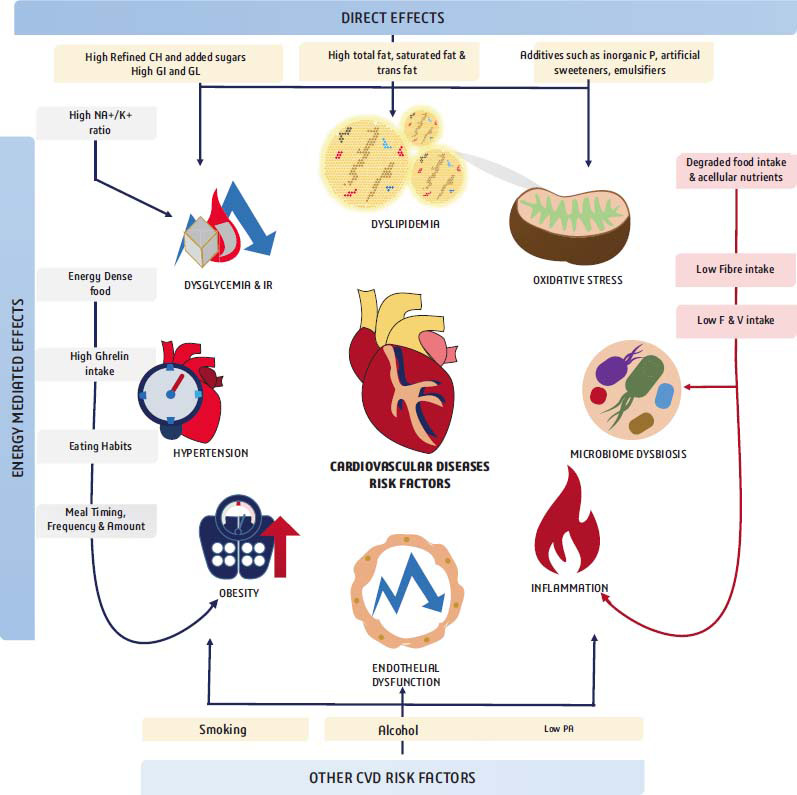
Exploring the potential biological pathways connecting dietary choices with cardiovascular disease.

Furthermore, the majority of cardiovascular risk factors promote endothelial dysfunction and damage while maintaining a prothrombotic and proinflammatory molecular environment. This intricate interplay results in a network of molecular feedback loops that drive and perpetuate atherogenesis, leading to a variety of cardiovascular disorders [22, 24, 25]. (Fig. **1**) describes the potential mechanism of cardiovascular complications connected to our food choices.

In epidemiological and experimental research, higher consumption of these types of foods has been linked to excess adiposity. Notably, Hall *et al.* discovered that as compared to a moderately processed diet, a high processed diet increased ad libitum energy consumption by 500 kcal/d and resulted in weight gain when compared to a moderately processed diet.

Over 9 and 4 years of follow-up, adult participants in the highest quartile of ultra-processed food intake had a 26-29 percent higher risk of becoming overweight in the prospective Seguimiento University of Navarra study and the Brazilian Longitudinal Study of Adult Health, respectively HR ((95 percent CI): 1.26 (1.10, 1.45) and 1.29 (1.08, 1.45)) [26].

In three cross-sectional studies conducted in Brazil and the United States, participants with the highest *versus* lowest intake of ultra-processed foods (top *vs.* bottom quintile/quartile) had 31-48 percent higher odds of being overweight, 41-97 percent higher odds of being obese, and 41-62 percent higher odds of abdominal obesity (defined as waist circumference 88/102 cm for men and women, respectively) [27]. The nutritional content of ultra-processed foods, as well as their ability to replace healthy, low-calorie meals from the diet, can be ascribed to their contribution to weight gain. The intense flavouring of ultra-processed items, which is caused by excessive quantities of fat, salt, sugar, and chemical additives, makes them highly palatable and may interfere with natural satiety systems. These foods are generally more energy-dense and less filling than minimally processed foods and prepared meals. The Oro-sensory properties of ultra-processed meals, such as their softer and less fibrous textures, allow for faster ingestion and may result in a higher energy intake in a shorter period. According to research, increasing eating rates can lead to overeating due to delayed satiety signalling. Furthermore, the convenience, cost, broad availability, and persuasive marketing of ultra-processed foods might encourage bad dietary habits, excessive snacking, and overeating, all of which contribute to increased calorie intake. Visual signals and food advertising depicting appetizing foods can trigger brain regions associated with overeating, leading people to eat even when not hungry. These variables, taken combined, highlight how the use of ultra-processed meals may contribute to excessive energy intake and subsequent weight gain [26, 28-31].

Excessive consumption of sodium is associated with high precedence of hypertension, a major factor in cardiovascular disease (CVD) and stroke. There is a link between increased sodium consumption, and elevated blood pressure involves various complex pathways, including disruptions in renal sodium balance, extrarenal sodium regulation, direct effects on blood vessels, and neuro-hormonal responses, particularly in individuals with salt-sensitivity phenotypes [32-34] These pathways cause metabolic, hemodynamic, and inflammatory alterations, resulting in volume expansion, water retention, endothelial stiffness, increased peripheral resistance, and higher blood pressure. A greater sodium-to-potassium ratio (1.0) has been connected to an increased risk of CVD mortality, whereas a higher potassium consumption has been linked to a decreased risk. Commercially processed foods are the predominant source of salt in the American diet. In contrast, potassium-rich, minimally processed foods such as milk, fruits, and vegetables are the key dietary sources of potassium (He *et al.*, 2020). A move towards more ultra-processed foods and less potassium-rich, minimally processed foods may result in increased salt consumption and an unfavourable sodium-to-potassium ratio, thereby increasing CVD risk [35, 36].

With the widespread use of inorganic phosphate salts as additives in industrial food processing, there has been concern about the cumulative phosphorus consumption in the US diet. When compared to organic phosphorus present naturally in meals (40-60%), inorganic phosphate is absorbed more efficiently (80-100%). High dietary phosphorus levels have been linked to cardiovascular disease (CVD) in both epidemiological and experimental investigations. This relationship is caused by changes in the hormonal control of extracellular phosphate, which results in Increased parathyroid hormone and fibroblast growth hormone release, both of which induce arterial calcification. Phosphate excess has also been associated with endothelial cell oxidative stress and impaired endothelial function [37]. Endocrine-disrupting chemicals such as bisphenol A may be present in industrially manufactured meals in elaborately wrapped containers (BPA). According to research, increased BPA exposure may raise the likelihood of key CVD risk factors such as diabetes, obesity, and hypertension [38]. BPA has been proven to promote insulin resistance, oxidative stress, inflammation, adipogenesis, and pancreatic B-cell dysfunction *via* binding to estrogen-related receptors. BPA is primarily absorbed through foods kept or reheated in BPA-lined containers. Although diets high in fresh and minimally processed foods are related to lower urine BPA levels, more research is needed to validate this. It is worth noting that one study discovered that higher consumption of sugar-sweetened beverages, rather than ultra-processed meals in general, was connected to higher urine BPA levels. Extensive heat exposure during food processing and preparation leads to the creation of advanced glycation end products (AGEs). These compounds have been associated with increased levels of oxidative stress and inflammation, and they may have a role in the development of cardiovascular disease (CVD). AGE production is especially prominent in animal meals heavy in protein and fat, especially when exposed to higher temperatures, longer cooking periods, and low moisture levels. Furthermore, dry-heat processing and deep-frying of carbohydrate-rich meals, including crackers, chips, cookies, and french fries, accelerate the formation of AGEs. However, an in-depth understanding of the role of dietary AGEs in the development of CVD and the causative link between AGEs and CVD is still lacking [38-43].

The gut microbiota is modulated by diet, and changes in microbial composition and intestinal barrier function have been linked to various health issues. Diets high in fiber, such as those rich in minimally processed plant foods, may boost microbial gene diversity and the formation of SCFA fermentation byproducts, which can affect host metabolism and immune system function. Low-fiber diets, on the other hand, shift gut microbial metabolism toward the use of proteins and host mucins, resulting in intestinal mucus layer disintegration and increased vulnerability to chronic inflammatory disorders [44, 45]. Changes in the physical shape of the food matrix during processing can potentially affect the makeup, metabolism, and development of the gut microbiota. Ultra-processed meals, which are frequently acellular and high in nutrients, may develop an inflammatory gut microbiota linked to cardiometabolic disorders. Additionally, consumption of low-calorie sweeteners and emulsifiers used in food processing may affect gut microbiota richness and balance, promoting metabolic diseases and insulin resistance [44, 45].

Based on the current strength and level of evidence, the American College of Cardiology/American Heart Association Guideline on the Primary Prevention of Cardiovascular Disease recommends avoiding trans fats and limiting intake of certain ultra-processed foods, such as processed red meats, refined grain products, and sweetened beverages. Despite this advice, many health professionals and doctors are still unaware of the potential health concerns connected with a wide range of ultra-processed meals other than the goods specified. A significant obstacle in the field of cardiovascular nutrition is the lack of extensive randomized clinical trials (RCTs) that directly measure concrete cardiovascular outcomes. Therefore, to establish a cause-and-effect relationship, evidence from well-designed perspective observational studies becomes crucial. These studies need to be supported by biological plausibility, meaning that the observed associations between dietary factors and cardiovascular health align with our understanding of physiological mechanisms. Preventive cardiology relies on comprehending how different food ingredients and nutritional patterns, collectively referred to as the human “food-ome,” exert their effects on cardiovascular health. This understanding of underlying mechanisms is essential both in theory and practical applications to promote heart health and prevent cardiovascular diseases [46].

### 3.2 Sedentary Lifestyle

A sedentary lifestyle is characterized by engaging in activities with minimal energy expenditure, typically 1.5 metabolic equivalent tasks (MET) or less, as defined by the Sedentary Behavior Research Network in 2012. These sedentary behaviors encompass activities such as watching television, playing video games, sitting at school or work, and commuting. Epidemiological evidence suggests that prolonged time spent in such sedentary behaviors can have various adverse effects on the human body through multiple mechanisms. One of the key impacts of sedentary behavior is its detrimental effect on metabolism. It has been observed to reduce lipoprotein lipase activity, impair lipid metabolism, and diminish carbohydrate metabolism. Additionally, sedentary behaviors decrease systemic blood flow while activating the sympathetic nervous system, resulting in reduced insulin sensitivity and vascular function, ultimately leading to decreased cardiac output. Such increased inactivity and sedentary time also contribute to weight gain, obesity, and heightened chronic inflammation in the body. In light of this, a cross-sectional study investigating the relationship between sedentary behavior and cardiovascular risk factors found that sedentary time had a harmful linear association with increased waist circumference, insulin resistance, and c-reactive protein levels. The strongest correlation was observed with triglycerides and markers of insulin resistance, which can be attributed to the mechanisms underlying sedentary behavior. Fewer skeletal contractions during sedentary activities reduce lipoprotein lipase activity, impair plasma triglyceride and glucose clearance, and decrease glucose-induced insulin secretion [47]. These studies highlight how sedentary behavior can influence several metabolic enzymes, leading to metabolic disorders.

Subsequent epidemiological studies have reported the relationship between sedentary behaviour and health outcomes. The meta-analysis of prospective cohort in the landmark study reports a significant positive association with moderate to vigorous physical exercise between sedentary time and all-cause mortality [48]. The finding from the landmark study was further validated by a systemic review which suggested an increased risk of about 147% in cardiovascular disease and 112% in type 2 diabetes and certain cancers associated with prolonged sedentary behaviour. From the meta-analysis, the incidence of cancer is associated specifically with breast, colon, endometrial, and epithelial ovarian cancer [49] When compared to uninterrupted sitting, breaking up prolonged sitting with light and moderate-intensity activity breaks was related to reduced systolic blood pressure (light: 120 1mmHg (calculated marginal mean SEM), *P*=0.002; moderate: 121 1mmHg, *P*=0.02) (123 1mmHg). While compared to continuous sitting, diastolic blood pressure was significantly lower during both exercise conditions (light: 76 1mmHg, *P*=0.006; moderate: 77 1mmHg, *P*=0.03) (79 1mmHg).

Emerging evidence points towards a positive relationship between sedentary behaviour with high blood pressure, independent of other risk factors like age, alcohol intake, and smoking. Hypertension also increases with increased exposure to sedentary behaviours [50, 51]. A cross-sectional study was conducted in periurban Uganda among 310 adults aged around 35 years to investigate the prevalence of a sedentary lifestyle with hypertension. The study reports 24.5% systolic hypertension and 31% diastolic hypertension, with 31% and 9% of obesity and diabetes, respectively, among the participants. Moreover, the study concludes the strong prevalence of a sedentary lifestyle with hypertension causes detrimental effects on health [52]. An experimental cross over trial was conducted to understand the cardiovascular response towards uninterrupted prolonged sitting. The cross-over study involved adults from the age of 19-65 years old who underwent three conditions: 1) uninterrupted sitting; 2) sitting with 2 minutes break of light intensity of walking at 3.2km/hr every 20 minutes; and 3) sitting with 2 minutes break of moderate intensity walking between 5.8-6.4km/hr every 20 minutes. Prolonged sitting resulted in impaired endothelial function with an increase in the mean arterial pressure. However, bouts of light and moderate intensity breaks resulted in lower systolic blood pressure of about 120 ± 1mmHg compared to the uninterrupted sitting of 123 ± 1mmHg. Similar confounding results were found in diastolic blood pressure with light intensity breaks: 76 ± 1mmHg, moderate intensity breaks: 77 ± 1mmHg compared to uninterrupted sitting 79 ± 1mmHg.

A comprehensive study was conducted on the low/mid-income populations from six different countries like, China, Ghana, India, Mexico, Russia, and South-Africa, to assess the impact of sedentary behaviour on population and health outcomes. According to the study, the prevalence of high sedentary behavior is approximately 8.3 percent, with the following country figures: China 9.0 percent (7.5-10.9 percent), Ghana 6.4 percent (5.1-8.1 percent), India 5.2 percent (4.2-6.4 percent), Mexico 3.9 percent (2.3-6.5 percent), Russia 17.7 percent (11.6-25.9 percent), and South Africa 4.6 percent (2.2-9.4 percent). Being jobless and living in a city are the most sociodemographic factors associated with sedentary behaviour in the whole sample. Sedentary behavior has also been linked to an increase in the frequency of chronic illnesses. Worsen health status involving low mobility, pain/discomfort, affected cognition, and sleep/energy were significantly correlated with higher sedentary behaviour [53]. Furthermore, there was a large-scale study conducted using data from the accelerometer, which found a dose-related response towards sedentary time and mortality risk factor; every 30-minute increment in the sedentary time consistent with a 17% higher risk of death [54].

Sedentary behavior and its link with metabolic illnesses have grown in prevalence as a result of urbanization and lifestyle changes. The growing prevalence of metabolic disorders corresponding to a sedentary lifestyle continues to pose a significant public health concern. Individuals with metabolic syndrome face an increased risk of diabetes, cardiovascular disease mortality, and greater occurrences of cardiovascular events and strokes. Understanding the link between sedentary behavior and metabolic diseases is critical for successfully addressing these public health concerns.

Potential processes that govern the link between sedentary behavior and metabolic risk factors are still in infancy. Still, there are several reports have been submitted to illustrate a few mechanisms involved. For example, reduced muscle movements in sedentary behaviour are common and have been subjected to a significant reduction in muscle lipoprotein lipase activity, an enzyme responsible for regulating lipid metabolism [55, 56] and, resulting in sustained energy imbalance and expansion of adipose tissue. After a brief period of immobilisation of the legs of the rats, investigators observed a significant reduction in lipoprotein lipase inactivity in skeletal muscles. In fact, immobilisation has been shown to lower lipoprotein lipase activity to 10% of its actual function in muscle fibres [57]. The consequence of glucose spikes with sustained energy imbalance can promote oxidative stress, endothelial dysfunction, and a biochemical inflammatory cascade reaction. As a result of this condition, there is a metabolic dysfunction that provides an environment for the growth of atherosclerosis and CVD [58].

### 3.3 Smoking

Smoking is a lifestyle factor. It has an intricate relationship among individuals and its profound effects on their health outcomes. Tobacco use increases mortality from all causes and has a major role in the development of atherosclerosis and cardiovascular disease [ASCVD]. Active smoking and second hand smoking account for around 30% of coronary heart disease mortality. Furthermore, cardiovascular diseases have become a major source of disease and mortality all around the world. A large portion of this load is attributable to a lack of knowledge and preventative initiatives, as well as inadequate control of atherosclerosis and cardiovascular illnesses (ASCVD). According to the World Health Organization estimates, smoking accounts for around 10% of all CVD cases. Tobacco consumption kills 8 million people globally and more than 7 million deaths result from direct smoking [59]. Smoking elicits detrimental damage to endothelial function while upregulating several oxidative mechanisms that impair platelet function, inflammation, and vasomotor function. Although epidemiological studies have convincingly shown the link between smoking and CVD, the particular mechanisms behind this relationship remain unknown. Cigarette smoking is the most complicated CVD risk factor because of the nature of its chemical components that are either stuck to aerosol particles or free in the gaseous phase. Fowles *et al.* have studied the association of a few chemical compounds towards toxicity and chronic diseases. They found that 1,3-butadiene is associated with cancer risk, and arsenic, cresols to cardiovascular disease [60].

Nicotianatabacum plant can absorb metals and this ability is used to decontaminate the soil. However, the presence of heavy metals like copper, mercury, aluminum lead, nickel, and zinc in tobacco products like cigarette smoke makes it more dangerous and toxic. Evidence suggests that cigarette smoke changes metal homeostasis, which may lead to chronic illness. A study found that metals in cigarette smoke have a critical role in disrupting vascular endothelial function [61, 62]. It accelerates the rate of response, resulting in oxidative stress and inflammation, which is the root cause of the rise in noncommunicable chronic diseases such as cardiovascular disease, cancer, and degenerative diseases. The aryl hydrocarbon receptor is liganded by polycyclic aromatic hydrocarbons found in cigarette smoke. The binding of such ligands to aryl hydrocarbon receptors accelerates the atherosclerosis process in apolipoprotein E deficient mice [63]. Furthermore, the accumulation of cholesterol in the macrophages due to cigarette smoke and chemotactic receptor CXCR-2 plays a relevant role in the process of inflammatory diseases, including atherosclerosis [64]. Evidence reports that continuous smoking harms the Flow of Mediated Dilatation (FMD) of the brachial artery and the effect is found to be dose-dependent [65]. After continuous smoking, it was found that there was a deleterious effect of reduction on the endothelial-dependent dilatation in coronary arteries, and it was reversible [66]. Therefore, cessation of smoking results in a significant reversal of FMD. Several pathogenic mechanisms of smoking-induced cardiovascular illness were outlined in the Surgeon General report from 2004: 1) endothelial damage, 2) prothrombic effect, 3) inflammation, 4) abnormal lipid metabolism, 5) risen myocardial oxygen and blood demand, 6) reduced myocardial blood and oxygen supply [67]. Nicotine present in the cigarette seems to address its effect by stimulation of the nicotinic acetylcholine receptors (nAChRs), present around the central nervous system and in other organs responsible for the parasympathetic autonomic nervous system. The adrenergic effects of nicotine result in raised heart rates, intropic status, coronary microvascular resistance, and impaired insulin sensitivity, consequentially responsible for the increased cardiovascular risks [68] Endothelium dysfunction impairs endothelial-dependent vasodilation, a process that needs nitric oxide. Smokers have lower nitric oxide bioavailability due to the existence of free radicals in the gaseous phase of smoking, which terminates the super oxide anions [69]. Many studies advocate that oxidant compounds from smoke are responsible for endothelial dysfunction and also lead to higher concentrations of extracellular lipid content resulting in the progression of the atherosclerotic process. Intracellular accumulation of lipids results in the expression of toll-like receptors in adipocytes and macrophages, inducing the activation of p38 and MAPK pathways. Insulin resistance in itself worsens the obesity status. Although both passive and active smokers have impaired vasodilatation, it seems to be worse on passive smokers [70]. Several studies have shown that smoking is a recognized risk factor for ASCVD; nevertheless, the negative health effects of passive smoke exposure are typically neglected. Human studies demonstrate that even brief exposure to second-hand smoke raises the incidence of acute myocardial infarction (AMI) by 30 percent. This has been connected mechanistically to increased platelet activity, decreased endothelial function, and increased inflammation/oxidative stress.

Higher the alterations in lifestyles, the risk factors have more influence on attenuating the risks associated with cardiovascular diseases. The deleterious effects of smoking and the health benefits of smoking cessation are well established. Evidence from the investigation on human studies observed that small-time exposure of smoke can increase the events of acute myocardial infraction by 30%. In this process, it has been coupled with increased platelet activity with an impaired endothelial function, increased insulin resistance linked with decreased energy metabolism, and increased oxidative stress. Second hand smoking (SHS) increases platelet activation, which thereby increases the activity of thrombus formation and damages the lining of arteries, which ultimately facilitates the development of atherosclerosis. In a study, it has been observed that second hand smokers (SHS) had a greater level of fibrinogen, which Is a passive mediator of platelet activation and an inflammatory marker linked to an increased risk of heart disease. In a Japanese study, they found that SHS had an 11.2 ± 4.1-mg/dL (mean ± SE) higher mean fibrinogen level than non-exposed non-smokers [71].

Cessation of smoking reduces the mortality rate by approximately one-third, a significant gain. Quitting smoking has been reported to reduce the incidence of cardiovascular events in the population; however, many individuals continue to smoke. In addition, the percentage of smoking-related deaths is increasing in many regions and populations. Therefore, it is essential to conduct additional research and policies to improve and reduce the habit of smoking.

### 3.4 Genetics

Obesity is a multifactorial and heterogenous condition with a close relationship to genetics. Recent advancement in obesity research reports the intricate molecular mechanism contributing to obesity. Thereby, playing a significant role in influencing the susceptibility of a person towards weight gain, obesity, and overall health. Within genetic etiology, they’re three kinds of obesity are considered: monogenic, syndromic, and common obesity. Within monogenic forms of obesity, the gene responsible for the phenotype is clearly observed; however, for common obesity, the loci architecture responsible for the phenotype is still under characterization. Moreover, the impact of genetic variation extends to metabolic processes, appetite regulation, and fat storage control, synergizing with lifestyle and dietary choices to contribute to the development of obesity. Furthermore, the understanding of genetic variation in metabolic processes intersects with the historical context of our ancestors' hunter-gatherer lifestyle, marked by limited food resources and the evolutionary preference for fat storage.

Over an extensive prehistoric time, our ancestors primarily lived with limited food resources, which resulted in favoring individuals capable of fat storage for energy. In modern societies, where food is abundantly present, leads to a shift in the natural selection process. However, our genetic make-up remains the same from early agricultural development about 12,000 years ago [72]. This lag in the genetic adaptation to swift changes in our modern societies consequently, the reason why many people gain weight effortlessly. Nevertheless, across various societies and subgroups, there exist both obese and non-obese individuals. This distinction primarily emerges as a consequence of genetic factors, as demonstrated substantial heritability observed for body mass index (BMI) in familial studies conducted by various researchers [73-75].

Familial studies show that children's BMI is highly proportional to parental obesity [76]. However, in familial studies, sometimes it is hard to distinguish the result of obesity from the consequence of genetic drift or environment. To address this question, a study was conducted about twin pairs or adopted children sharing information about genetic influence on BMI. A recent meta-analysis of 31 twin studies shows that genetic differences from 47% to 80% explain the adult's BMI variation [77]. In another study by Silventoinen and colleagues where analyzed 87782 twin pairs from 45 cohorts to summarise the role of genetic factors in the variation of BMI [78]. An adoption study demonstrates that the BMI of adopted children is strongly inclined toward the biological parents [79]. This study concludes the relationship between genetics and their contribution to variation in BMI. Several studies have surfaced, discovering 100 BMI- BMI-associated loci when the sample is compared among obese and non-obese people. Studies established the role of genes contributing to the role of genes towards proinflammation associated with the severity of atherosclerosis. Furthermore, there are some genes found to be responsible for causing obesity in a study involving rodents [80], which are now also considered to be contributors to severe obesity in humans [81]. Nonsyndromic monogenic forms of obesity result from mutations in a single gene, affecting approximately ~5% of the population. These rare mutations create a loss of function or deficiency in food intake and energy homeostasis. Most of these mutations occur in LEPR (Leptin receptor), Melanocortin 4 receptor (MC4R), and pro-opiomelanocortin (POMC) genes. The results from a study show that a mutation or deletion in the POMC gene with an allele frequency of 12% affected overall body weight and food motivation in labrador retrievers. This study extrapolates the importance of the leptin and melanocortin pathway on obese phenotype [82]. Although nonsyndromic monogenic forms of obesity exist in our society, most obesity results from food abundance and lack of physical activity. The initial technique employed to identify the gene associated with common obesity was the candidate gene association study method, outlined by Day *et al.*, 2011 [83]. In obesity research, the primal focus is on polymorphism and the linked regions responsible for coding proteins involved in the regulation of lipid and glucose metabolism, food consumption, and energy expenditure [83-85]. However, the limitation of this approach is inadequate information on the molecular mechanism of these genes associated with the disease. The genetic consequence of obesity resulting from a sedentary lifestyle resolved at a higher resolution with the completion of the human genome project. The completion of the human genome project led to the discovery of the first locus, to be associated with obesity was insulin-induced gene 2 (INSIG2) [86]. Furthermore, there were subsequent discoveries through genome wide association studies (GWA) was the FTO gene [87]. Initially, the GWA study was conducted to test the relationship between the polymorphism across the human genome and type 2 diabetes (T2D). The study reported the rs9939609 polymorphism present in the first intron region of the FTO gene, which is strongly associated with type 2 diabetes and increased BMI. Other polymorphisms in the intronic region of the FTO region was also consistently associated with severe early onset of childhood and adult obesity (rs1421085 and rs17817449) and the association was extended to other obesity-related traits such as bodyweight and waist-to-hip circumference ratio (rs9930506) [88, 89]. FTO polymorphism has been linked further to abdominal obesity, waist circumference, and body fat percentage [90-92]. With the surge in cases of obesity in our society and the advantages of our technology, it has become imperative to understand the genetic correlation between genes and their nature to influence the mechanism of energy homeostasis, causing variation in body weight at any given location. Hypertension, obesity, and dyslipidemia have become the predictive markers to project and development of cardiovascular disease (CVD) appropriately. These risk factors can influence the projectile of cardiovascular risk and its prognosis. In addition, these risk factors have some genetic component, but these diseases are genetically complex, and a tiny portion of these are accounted under Mendelian forms. Therefore, identifying the genetic determinants of this disease could allow us to intervene in this epidemic of obesity.

### 3.5 Environment

Obesity results from pleiotropic interaction between diet, physical activity, and the environment. The environment can progress and stimulate the elements that make up the structure that influence obesity, which are obesogenic elements. Recent reviews describe the role of obesogenic elements in promoting obesogenic progress and chronic low-grade inflammation. These obesogenic elements are reactions favoured by the lifestyle and urban design that promotes the use of a hypercaloric diet. One model describes obesity is the normal reaction response associated with an unfavourable environment [93]. The model describes how biological, age, sex, and environment come under the macroenvironmental factors to influence obesity, while behavioural, emotions, beliefs, cognition, and habits come under the microenvironmental factors to influence obesity. Macroenvironment factors can influence obesity in the population. However, microenvironment factors can influence obesity among individuals. Obesogenic elements have been reported to add changes in the lifestyle, which involve lower physical activity, mostly in urban areas [94]. Organisms living in an obesogenic environment become inflamed, which promotes an immune response known an chronic low-grade inflammation or systemic inflammation. Adipose tissue in an obese organism shows constant inflammation as well as increased migration of macrophages [95]. These changes result in chronic low-grade inflammation and insulin resistance. Adipose tissue is reported to be the primary source of adipokines, which are cytokines, chemokines, and metabolic mediators. During obesity, these adipokines, along with tumor necrosis factor-alpha, interleukin [IL] - 1β, 6, 18, are found with increased serum levels [96, 97]. However, these factors aren’t only responsible for obesity. Environment pollution is shown to directly influence a person's health through direct pathological impacts of various chemicals, physical and biological agents, and indirect approaches through promoting physical and social activity. Understanding the impact of the environment on obesity and its related risk factors could provide the information necessary to prevent the epidemic of obesity. Environmental pollution constitutes a significant amount of death and its relationship to developing diseases. Exposure to some environmental pollutants is reported to be responsible for the obesogenic process; these are called obesogenic pollutants. The substances are produced mainly by combustion, pesticides, organic pollutants, and heavy metals. And the diet is the principle source of exposure to these substances [98, 99]. Pollution in the environment favours the mechanism of developing chronic low-grade inflammation and obesity. Chronic low-grade inflammation operates through different pathways like, the presence of peroxisome proliferators, which are nuclear hormone receptors linked to be associated with inflammation, adipogenesis, and lipid metabolism. Chronic low-grade inflammation also causes thyroid and adipogenic tissue imbalance, as well as immune system harm [100, 101].

Other studies suggest that the recent boom in the incidence of obesity is primarily due to environmental factors, which include high food consumption, sweetened beverages, and less activity [102]. Due to socialization and living in industrialized states, we enjoy exposure to high-fat/caloric diets. Furthermore, our demands for physical activities have changed. The modern states of living introduced to obesogenic environments. Several studies have identified obesogens that can stimulate the risk of obesity. These obesogens have been identified in blood and urine. The concentration of metabolites of bisphenol A and phthalates – byproducts of plastic and other consumer goods in the urine is associated with a greater risk of weight gain by interacting with the endocrine disruptors [103]. People from the previous century adhering to traditional diets and lifestyles have reported to have lesser obesity rates compared to people living in urbanized or Western-influenced environment. In this study, the researcher examined the dietary habits and lifestyle habits of the people from the Inuit of Canada and the Sami of Norway. The researchers found that indigenous people from these places consumed local traditional cuisines, which are nutrient-dense food like fish and plant-based sources, which reduced the obesity rates compared to their urban counterparts. The study emphasizes the prevention of obesity in non-obesogenic situations through traditional meals and behaviours [104]. In another study where the researcher compared the physical activity patterns among urban and rural populations in India. The researchers found that people residing in rural areas tend to have an agrarian lifestyle and higher engagement in manual labour, increasing the level of physical activity in comparison to urban dwellers. These differences in physical activity result in lower rates of obesity among rural people, emphasizing the impact of a non-obesogenic environment that promotes a higher level of physical activity, thereby reducing obesity related risk factors [105]. These examples advocate the influence of non-obesogenic environments on obesity rates and lifestyle factors. These emphasize the significant role of traditional diet, physical activity, and lifestyle towards contributing an healthy outcomes in different populations. The researchers and policymakers can understand the silent differences in non-obesogenic environments to identify potential strategies for promoting healthier outcomes and reducing the obesogenic settings.

## 4 THE LINK BETWEEN OBESITY AND CARDIOVASCULAR DISEASE

**Fig. (2):**
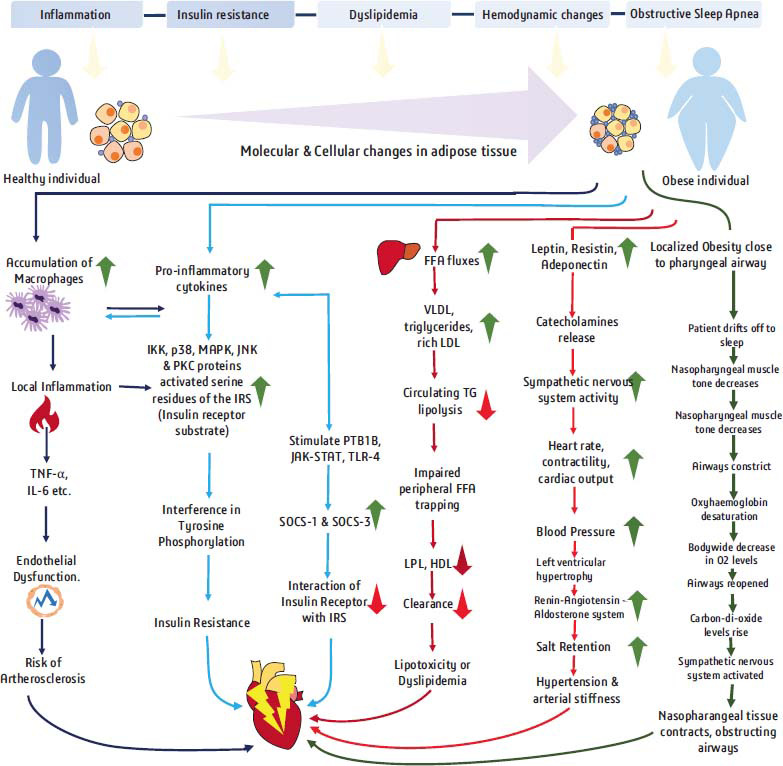
Visual depiction unveiling how obesity drives cardiovascular disease progression: A schematic overview.

Obesity is becoming more common around the world. A number of epidemiological investigations have found that obesity has a significant role in the development of cancer, cardiovascular disease, type 2 diabetes, liver disease, and other ailments, imposing a substantial burden on the public and health-care systems each year. Excess energy intake causes adipocyte hypertrophy, hyperplasia, and the production of visceral fat in non-adipose tissues, resulting in cardiovascular and liver illness. Adipokines and inflammatory cytokines can also be secreted by adipose tissue to impact the regional microenvironment, promote diabetes and insulin resistance, hyperglycemia, and activate related inflammatory signaling pathways. This aggravates the development and progression of obesity-related disorders. Following are the pathophysiological mechanisms involved in the development of CVD in obese patients. (Fig. **2**) gives a detailed view of the pathophysiological mechanism of obesity-induced cardiovascular complications.

### 4.1 Inflammation

Adipose tissue has now been understood to be a functional endocrine organ that performs a variety of functions in the body rather than just a fat storage organ. Both vascular and nonvascular tissues in obese people with adipose tissue are involved in the inflammatory process. Unusual concentrations of metabolites from adipose tissue, including lipids, fatty acids, and cytokines, activate monocytes and stimulate the release of inflammatory cytokines. Obese people's adipose tissue has active macrophages, which work with adipocytes to release a variety of cytokines [3]. Vasoactive substances like leptin, adiponectin, tumour necrosis factor-alpha (TNF-), interleukin-1 (IL-1), interleukin-6 (IL-6), and other inflammation-related adipokines, PAI-1, angiotensinogen, and endothelin, as well as molecules that may contribute to insulin resistance like FFA, TNF- and resistin, are among them which participate in the inflammation process and affect metabolic function and might play a role in causing cardiovascular end-organ damage. The type I Interleukin 1 receptor (IL-1R/IL-1R1), a Toll-like receptor that heterodimerizes with the IL-1R accessory protein, is involved in IL-1 signaling (IL-1RAcP). An anti-inflammatory cytokine called interleukin 1 receptor antagonist (IL-1Ra) binds to IL-1R in opposition to the proinflammatory cytokine IL-1. Inflammatory signalling is either “on” or “silenced,” depending on how much of the IL-1R1-IL-1RAcP receptor complex is occupied by either an IL-1 agonist or an IL-1Ra. So, that is where the primary mechanisms of inflammation begin. Adipose tissue changes at the molecular and cellular levels as people gain weight and their adipocytes grow, which has an impact on systemic metabolism. Adipose tissue contains an accumulation of macrophages that first causes local inflammation. Adipose tissue from obese people expresses proinflammatory proteins, including TNF- and IL-6 more than that of lean people. Adipose tissue produces a number of proinflammatory substances as obesity levels rise. Obesity also raises the number of macrophages in adipose tissue, which reportedly act as scavengers of apoptotic adipocytes. Moreover, it has been noted that these scavengers are significantly more prevalent in obese people. Many metabolic disorders associated with obesity, such as atherosclerosis and systemic inflammation, are thought to be caused by macrophage accumulation and the accompanying local inflammation. More cytokines are released by visceral fat than by subcutaneous adipose tissue. This systematic inflammation leads to endothelial dysfunction, clinical and experimental studies support the connection between systemic inflammation and endothelial dysfunction. There is growing data that suggests that abnormal endothelial function may act as an early indicator of an active atherosclerosis disease. As a result, endothelial dysfunction has come to be understood as having a significant impact on a number of illnesses linked to a high prevalence of atherosclerotic CVD. The production of inflammatory cytokines, which have an impact on the entire atherosclerotic vessel, is a key factor in the development of atherosclerotic plaques. It is significant to note that the impairment of endothelial cells' normal function characterizes the formation of atherosclerotic lesions, regardless of risk factors (such as diabetes, hypertension, and obesity) [1, 44, 106-109].

### 4.2 Insulin Resistant

The relationship between obesity and a reduction in insulin-mediated glucose uptake has been well-established for more than 3 decades. The connection between fat and insulin resistance, however, is still debatable on a number of fronts despite its lengthy history. It is generally believed that metabolic disorders like insulin resistance and obesity go hand in hand. If not, it would be impossible to comprehend how the idea of a metabolically obese, normal-weight person came to be. This idea is evidently founded on the notion that insulin resistance and metabolic abnormalities are the usual states of fat people and that nonobese people rarely exhibit these findings. The finding that insulin resistance is not solely a result of being overweight or obese is perhaps the most compelling. As a result, type 2 diabetes is a common related illness that affects 40% of fat people. A 5% decrease in baseline body weight was linked to a 60% reduction in the risk of new-onset diabetes, as compared to subjects who did not lose weight, according to the Diabetes Prevention Study, which examined the effects of lifestyle modification on the risk of developing diabetes. Consistently, a 7% body weight loss in the Diabetes Prevention Programme was linked to a 58% decreased chance of acquiring diabetes at a 4-year follow-up [110].

Obese fat tissue releases a variety of pro-inflammatory adipokines, including monocyte chemotactic protein-1 (MCP-1), tumour necrosis factor-alpha (TNF-alpha), interleukin 1 beta (IL-1), and interleukin 6 (IL-6). By attracting C-C motif chemokine receptor 2 (CCR2) to fatty adipose tissues, secreted MCP-1 attracts monocytes during the chemotaxis process. Monocytes develop into macrophages once they unite with adipose tissues at the location of the inflammation. Adipose tissue macrophages (ATMs) that are local to an obese person polarise from an anti-inflammatory M2 to a pro-inflammatory M1 phenotype. Depending on the size and condition of the adipocyte, resident pro-inflammatory M1 macrophages release cytokines such as MCP1, IL-1, and IL6, which might attract additional monocytes. The M1 macrophages and adipocytes in obese adipose tissues emit excessive amounts of pro-inflammatory cytokines, which attract additional monocytes. The percentage of macrophages can reach over 50%. Chronic overeating and obesity-related defective adipose tissues eventually cause FFAs, ROS, and pro-inflammation in the systemic milieu, initiating the first stage of low-grade systemic inflammation [107].

When there is an increase in systemic TNF- α and other proinflammatory cytokines owing to obesity, IKK, p38 MAPK, JNK, and PKC proteins are activated. These proteins directly target the serine residues of the insulin receptor substrate (IRS) protein and interfere with tyrosine phosphorylation, resulting in insulin resistance in the hepatocytes muscles and adipose tissues. Additionally, TNF- α stimulates PTP1B, which weakens insulin signalling by dephosphorylating phospho-tyrosine residues in the insulin receptor and IRS protein. Elevated IL6 level induces the JAK-STAT signalling pathways and the expression of SOCS1 (suppressor of cytokine signalling 1) and SOCS3. By altering the kinase activity, they sterically block the interaction of insulin receptor (IR) with IRS protein, downregulating insulin receptors' function. Additionally, IL-6 can activate STAT3 and trigger TLR-4 gene expression, and when combined with IL-1β, these two molecules increase the activity of STAT3 and NF-κB in hepatocytes, resulting in inflammation. By the phosphorylation on serine residue of IRS1/2, IL-1β inhibits the insulin signaling pathway by activation of p38 MAPK *via* the IL-1β receptor. There isn't much evidence that pro-inflammation dysregulates - β Cells, although the TNF- α may reduce β -cells' insulin sensitivity through nitric oxide [1, 4, 107].

### 4.3 Dyslipidaemia

The most important factor that leads to atherosclerosis and consequent cardiovascular disease (CVD) in obese people is likely dyslipidemia. The intricate connection between obesity and dyslipidemia is directly influenced by triglyceride levels, insulin resistance, and body fat distribution. Low-density lipoprotein cholesterol (LDL-c) and high-density lipoprotein cholesterol (HDL-c) in particular are altered, as are plasma triglycerides and cholesterol. Increased FFA fluxes from adipocytes to the liver and other tissues, decreased circulating TG lipolysis, impaired peripheral FFA trapping, and the production of small dense LDL are all part of the multifactorial pathophysiology of the typical dyslipidemia observed in obesity [111]. The most prevalent metabolic disease in obesity, insulin resistance/hyperinsulinemia, is the key factor contributing to the emergence of dyslipidemia. In recent years, the term “metabolic dyslipidemia” has been used to describe the type of dyslipidemia that results from the combined effect of insulin resistance and fat. Insulin's effects on lipid metabolism are well understood and well known. By inhibiting hormone-sensitive lipase (HSL), insulin prevents lipolysis in adipose tissue, thereby regulating the release of FFAs into the bloodstream. Also, Very low-density lipoproteins (VLDL) are secreted from the liver less often when insulin is present. Insulin also accelerates apoprotein B-100 (apoB100) breakdown. Insulin promotes the hydrolysis of TG from VLDL particles by hepatic lipase (HL) and lipoprotein lipase (LPL) in circulation, destroying TG-rich lipoproteins. Insulin stimulates the dephosphorylation of 3-hydroxy-3-methylglutaryl-CoA (HMG-CoA) reductase in the liver, activating the enzyme and speeding up the production of cholesterol. Hypertriglyceridemia develops when the plasma clearance of TG-rich lipoproteins is slowed down due to insulin resistance. In these conditions, the activity of the cholesteryl ester transfer protein (CETP) encourages the exchange of TG with cholesteryl esters between lipoprotein particles. Due to the increase in TG content, LDL and HDL particles shrink and become denser after being hydrolyzed by plasma lipases. Small, defective HDL particles and structurally altered LDL (sdLDL) HDL particles accumulate as a result of these functional effects [112, 113].

### 4.4 Hemodynamic Changes

Obesity is capable of producing a variety of hemodynamic alterations, which may predispose to changes in cardiac morphology and ventricular function. These alterations may contribute to the development of cardiovascular disease. They are most pronounced in severely obese individuals but may occur to a lesser extent in overweight and mildly to moderately obese adults, adolescents, and children. However, obesity-related changes in cardiac morphology and ventricular function have traditionally been attributed to hemodynamic alterations; recent experimental studies suggest that certain neurohormonal and metabolic abnormalities may contribute to obesity-related changes in cardiac structure and function [114]. These complicated and multivariate hemodynamic changes include variations in cardiac output, blood pressure, vascular resistance, and arterial stiffness. Increased cardiac output is one of the main ways that obesity affects hemodynamic. Leptin, resistin, and adiponectin are just a few of the cytokines and hormones that are known to be secreted by adipose tissue, particularly visceral fat, and which can have an immediate impact on the health of the heart. For instance, leptin can stimulate the release of catecholamines and increase sympathetic nervous system activity, which can both raise heart rate and contractility and raise cardiac output [115, 116].

Additionally, obesity and insulin resistance are frequently linked, and both of these factors can raise cardiac output. Blood pressure changes are another route through which obesity can cause hemodynamic abnormalities. Obese people are more prone to high blood pressure, which can raise the heart's afterload and eventually cause left ventricular hypertrophy. Increased sympathetic nervous system activity, activation of the renin-angiotensin-aldosterone system, and increased salt retention are regarded to be the main contributors to obesity-related hypertension. Obesity can also affect vascular resistance and arterial stiffness, which can raise the risk of CVD [117]. Particularly visceral fat is known to secrete pro-inflammatory cytokines, including tumour necrosis factor-alpha (TNF-alpha) and interleukin-6 (IL-6), which can impede vasodilation and contribute to endothelial dysfunction. Along with this, obesity is linked to increased oxidative stress, which can cause vascular damage and stiffness [117-120].

### 4.5 Obstructive Sleep Apnea

Obstructive sleep apnea (OSA) is a common breathing problem that affects people while they are asleep. The data supporting OSA's independent risk for the onset of hypertension, cardiovascular morbidity and mortality, and sudden death is accumulating. The pathophysiology of OSA can be complicated, multifaceted, and poorly understood. Obesity is one of several variables that could cause OSA. The distribution of fat is not uniform among obese people. BMI measures an individual's overall gain in body weight relative to their height and has a weak but significant link with the severity of OSA. In contrast to BMI, neck circumference shows localised obesity close to the pharyngeal airway and is correlated more strongly with OSA severity. Both the presence of OSA and the severity of OSA are associated with the volume of adipose tissue accumulated next to the pharyngeal airway. Greater significantly, compared to non-OSA people with BMI matches, obese OSA patients developed greater visceral adipose tissue [121]. Thickened lateral pharyngeal walls, nasal congestion, enlarged uvula, facial malformations, and tonsillar hypertrophy are also factors that contribute to OSA. As the patient drifts off to sleep, the nasopharynx's muscle tone decreases and its airways constrict. Repeated oxyhemoglobin desaturation, a body-wide decrease in oxygen levels, and brief micro-arousals by the patient are all common symptoms of these episodes. When the airway is reopened, carbon dioxide levels rise. As a result, the sympathetic nervous system is activated, and the nasopharyngeal tissue contracts, obstructing the airway [121, 122]. With a shorter duration of non-rapid eye movement (NREM) and Rapid eye movement (REM) sleep, these cyclical periods last the entirety of the night. Numerous studies have demonstrated that in OSA patients, the muscles that normally open the airways during inspiration are no longer able to withstand the negative pressure. The respiratory bouts can happen 50-100 times an hour in severe cases, with each event lasting 20-40 seconds. Obesity is one of the most significant reversible warning signs for obstructive sleep apnea syndrome (OSAS)-, accounting for 41% and 58% [123], respectively, of all cases and moderate-to-severe cases [124, 125].

## 5 POST COVID CARDIOVASCULAR COMPLICATIONS

The relationship between COVID-19 and obesity is a two-way street. Lockdown tactics implemented in the early stages of the pandemic to stop the spread of SARS-CoV-2 resulted in a considerable rise in the incidence of obesity, a condition termed “covibesity”. Conversely, a wealth of data has demonstrated that obesity is a determining factor for the severity of COVID-19 [109, 123, 126]. Obesity has been identified as a key prognostic factor for a severe course of COVID-19 in a study of 3,615 individuals. Compared to patients with normal body weight, patients aged 60 years with a BMI >30 kg/m^2^ had a 2-fold increased chance of being hospitalised and developing a serious illness (Huang *et al.*, 2020). In a different retrospective study, it was discovered that 75.8% of the 124 patients who were admitted to the intensive care unit (ICU) for severe COVID-19 were fat. A BMI >23 kg/m^2^ is related to a worse prognosis in patients affected by severe COVID-19, particularly in those aged 40 years and Black people, according to a prospective cohort study that included 7 million participants. A rise in BMI has been found to increase the likelihood of hospitalisations, mortality, and ICU admission. Patients with severe obesity (BMI >35 kg/m^2^) had a 4-fold greater probability of ICU admission [127].

In addition to viral pneumonia, COVID-19 also has a wide range of extra-pulmonary side effects, including cardiovascular and cerebrovascular illness. The sympathetic nervous system can be overstimulated by COVID-19, which can also result in a cytokine storm, hypercoagulopathy, and inflammation. Even after recovering from COVID-19, these pathways may cause permanent harm to the cardiovascular and respiratory systems [128]. The incidence of cardiovascular or cerebrovascular disease among COVID-19 survivors is anticipated to rise in the future as a result of these permanent sequelae, such as congestive heart failure or diminished lung function. The short-term cardiovascular outcomes in COVID-19 survivors have been covered in a few studies. Significant data from the National Healthcare Databases (VHA) of the US Department of Veterans Affairs (VHA) were recently reported by Xie *et al.*, who found that both hospitalised and non-hospitalized survivors of acute COVID-19 have a significant risk of CVD and a high 1-year burden of the disease [129] According to a study that was published in the Journal of the American Medical Association (JAMA), 19% of a cohort of 100 individuals with COVID-19 had signs of myocardial damage. The incidence of acute myocardial damage was 36.7%, according to a different study that was published in the European Heart Journal and involved patients hospitalized with COVID-19. A higher risk of thrombotic events, such as deep vein thrombosis and pulmonary embolism, has also been associated with COVID-19 [130, 131]. Below mentioned Fig. (**3**) shows the mechanism of how COVID-19 directly or indirectly affect cardiac health.

Based on research done during the past SARS, MERS, and COVID-19 epidemics, numerous causes for cardiac injury have been proposed. Cytokine release syndrome, a component of the systemic inflammatory response in severe COVID-19, can cause damage to numerous tissues, including cardiac myocytes and vascular endothelium. In severe cases, many proinflammatory cytokines, such as tumour necrosis factor (TNF)-, interleukin (IL)-2, IL-10, IL-6, and IL-8, are markedly increased acute respiratory distress [132] syndrome (ARDS) and other end-organ damage are caused by significant continuous inflammation (phase 2) and the critical role played by cytokines during viral infection (phase 1). Acute damage to the heart, the lung, and the endothelial cells may result from the interaction of SARS-CoV-2 with ACE2 [133]. A small number of case reports have suggested that SARS-CoV2 may cause viral myocarditis by directly infecting the myocardium. However, it appeared that in the majority of cases, myocardial damage was brought on by a rise in cardiometabolic demand brought on by systemic infection and persistent hypoxia brought on by severe pneumonia or ARDS. Cardiotoxic adverse effects are possible with some drugs, including corticosteroids, antiviral drugs, and immunological agents. Any serious systemic illness that affects the electrolytes can lead to arrhythmias, which puts patients with underlying heart disease at greater risk. Given the interaction of SARS-CoV-2 with the renin-angiotensin-aldosterone pathway, there is a specific concern regarding hypokalemia in patients with COVID-19 [5, 134]. It is well-recognized that hypokalemia makes people more susceptible to certain types of arrhythmias. These are the possible mechanism which covid-19 infection can cause cardiovascular damage in patients.

## 6 PREVENTIVE STRATEGIES FOR OBESITY INDUCED CARDIOVASCULAR DISEASE

In a world where delicious yet unhealthy food is available at every turn, it's no surprise that obesity has become a major issue. The “toxic food environment” we live in is making it harder and harder for our bodies to regulate weight through natural mechanisms. Lifestyle modifications have emerged as a viable solution for managing and preventing chronic diseases such as cardiovascular disease. Studies have shown that lifestyle changes, including increased physical activity, dietary adjustments, and stress reduction techniques, can have a significant impact on reducing the risk of chronic diseases [135]. These changes lead to weight loss and improve overall health habits, reduce comorbidities, limit negative side effects, and enhance one's quality of life. While there are pharmacological strategies available for weight loss, lifestyle interventions like the Diabetes Prevention Program have been proven to be just as effective. So don't give up on your health goals - with some determination and a commitment to a healthier lifestyle, you can achieve a better, more vibrant life. The main elements of a thorough lifestyle intervention are succinctly summarised below. Reviewing the Obesity Guidelines, the Diabetes Prevention Programme, the Look AHEAD (Action for Health in Diabetes) study or the Diabetes Prevention Program's treatment procedures can provide more in-depth information [136, 137].

### 6.1 Modification of Diet

In order to lower the risk of obesity and cardiovascular diseases, diet is essential. There are numerous ways that a healthy diet might offer these advantages. When calorie intake exceeds energy expenditure, there is an energy imbalance that leads to obesity. A balanced diet can successfully modify caloric intake, preventing excessive weight gain, by controlling the quantity and quality of food consumed [138]. In managing obesity, the macronutrient profile—which includes carbs, proteins, and fats—plays a crucial role. A diet high in whole grains, lean proteins, and healthy fats while simultaneously restricting the consumption of added sweets and saturated fats promotes optimal body weight regulation. Foods with a high energy density have a higher fat and sugar content and provide a significant caloric load per unit volume, which increases the likelihood of overeating. On the other hand, choosing low-energy-density meals like fruits, vegetables, and lean proteins might help you reduce your calorie intake while still getting enough nutrients. High-fiber foods, including whole grains, legumes, fruits, and vegetables, help regulate hunger and enhance fullness by slowing down blood glucose fluctuations through delayed digestion and nutrient absorption [139].

Some meals have the power to increase thermogenesis, the process through which the body burns calories by producing heat. For example, capsaicin in foods like chili peppers can speed up metabolism and help with weight management. The production and release of hormones linked to hunger regulation are also influenced by the food makeup. A high-protein diet can stimulate the production of more satiety hormones, whereas consuming too many sweet meals might upset the hormonal balance and lead to weight gain. Energy metabolism is substantially impacted by the gut microbiota, a complex ecosystem of microorganisms living in the gastrointestinal tract [140]. Prebiotic fibres, which can be found in fruits, vegetables, and whole grains, are certain dietary components that help the growth of good bacteria, possibly improving weight management. It is essential to understand that every person will react differently to dietary treatments, necessitating consultation with medical professionals or qualified dietitians to create a personalised and balanced diet plan suited to each person's needs and objectives. To effectively manage weight and reduce the risk of problems from obesity, maintaining a healthy diet calls for a long-term commitment and the establishment of sustainable eating habits [141, 142].

Numerous dietary strategies have been used over the years to regulate an individual's weight successfully. The MeD is a plant-centric nutritional diet that emphasizes excessive consumption of fruits, vegetables, legumes, nuts, seeds, and whole grain cereals. This is one of the best for CVD and weight management. The principal source of fats is olive oil. Red wine, eggs, dairy products, and shellfish are all acceptable food choices on the diet. Aside from that, the diet forbids the consumption of sweets and meats, particularly red meat [143, 144]. By increasing the diet of nuts and olive oil, the PREDIMED trial looked into how MeD might affect CVD. The study showed that a diet heavy in unsaturated fat was far better than a low-fat diet.

As a result, a 30% decrease in CVD risk was seen. Moreover, there is a rise in HDL and a modest drop in TG and LDL levels. In a similar vein, the PREDIMED-PLUS research focused on encouraging weight loss and exercise to reduce the risk further. According to another study, participants who followed MeD experienced a 25% drop in CVD risk. To better understand how diet lowers the risk of CVD, research is being done. According to the study findings, diabetes and metabolic syndromes are negatively linked with MeD. Additionally, it results in weight loss, a drop in TG, and LDL, and an increase in HDL, which significantly lowers the risk of atherosclerotic disease. MeD is advised as the primary method of preventing CVD because studies have demonstrated its effectiveness in reducing the chance of developing the disease. Furthermore, it has been shown that dietary changes, even those made later, can be beneficial. MeD has been shown to have a positive impact on BP [144-147]. Thus, MeD has been proven to be a great dietary preventative strategy that has long-term advantages in preventing insulin resistance development, inflammation, and glucose metabolism. Following a Mediterranean diet was linked to a lower risk of CVD, including coronary heart disease and stroke, according to a large prospective cohort study comprising more than 25,000 women [148].

The DASH diet allows for the consumption of fish, poultry, nuts, vegetables, fruits, cereals, and low-fat dairy products. Instead, it limits the consumption of processed meat, red meat, and sugar-sweetened beverages. It's encouraged to consume as little sodium and cholesterol as possible. It emphasizes the consumption of proteins and fibres, as well as minerals, including calcium, potassium, and magnesium. The DASH diet has been shown to be successful in lowering LDL levels. The NHLBI has promoted the DASH diet as a preventative measure against hypertension as a result of the encouraging results. The risk of high blood pressure and cardiovascular disease has impressively decreased as a result of consistent exercise, weight loss, and adherence to the DASH diet [149, 150].

### 6.2 Engage in Regular Physical Activity

Physical activity helps in obesity and cardiovascular management through various mechanisms. Here are some key mechanisms: Physical activity increases energy expenditure, leading to the burning of calories. Regular exercise helps create an energy deficit, which is essential for weight loss and weight management. By burning calories, physical activity can contribute to reducing excess body weight and preventing obesity. A recent study published in Obesity Reviews analyzed 203 articles and found that physical activity interventions significantly reduced body weight and waist circumference in adults with overweight or obesity [151]. Engaging in physical activity, especially strength training exercises, helps build lean muscle mass. Muscle tissue has a higher metabolic rate compared to fat tissue, meaning that having more muscle can increase your resting metabolic rate. This increased metabolic rate contributes to burning more calories even at rest, supporting weight management efforts. Physical activity enhances insulin sensitivity, which is the body's ability to respond to and utilize insulin effectively. Improved insulin sensitivity helps regulate blood sugar levels and reduces the risk of insulin resistance and type 2 diabetes. By managing insulin levels, physical activity can contribute to weight control and reduce the risk of cardiovascular diseases associated with obesity and diabetes [152]. Regular aerobic exercise improves cardiovascular fitness by strengthening the heart muscle and enhancing its efficiency. Physical activity increases heart rate, improves blood flow, and promotes the dilation of blood vessels, leading to improved cardiovascular function. Enhanced cardiovascular fitness lowers the risk of developing cardiovascular diseases such as coronary artery disease, stroke, and heart failure. A recent meta-analysis published in JAMA Cardiology reviewed 36 randomized clinical trials and concluded that exercise-based cardiac rehabilitation significantly reduced cardiovascular mortality and hospitalization [10]. In accordance with the World Obesity Federation's World Obesity Atlas 2023, if prevention and treatment approaches do not improve, the global economic burden of overweight and obesity will reach $4.32 trillion annually by 2035. This is similar to the impact of COVID-19 in 2020, which is estimated to be around 3% of the global GDP. If present trends continue, the majority of the world's population (51%, or more than 4 billion people) will be overweight or obese by 2035. Obesity will affect one in every four persons (almost two billion). If present trends continue, the majority of the world's population (51%, or more than 4 billion people) will be overweight or obese by 2035. Obesity will affect one in every four persons (almost two billion). In China, one research of 12,543 participants followed for 22 years found that the prevalence of age-adjusted obesity increased from 2.15% to 13.99% in both sexes, rising from 2.78 to 13.22% in females and 1.46 to 14.99% in males. Since 2000, the overweight rate of African children under the age of five has increased by 24%. In 2019, nearly half of Asian children under the age of five were obese or overweight. According to WHO data from Sub-Saharan Africa, the prevalence of overweight and obesity in adults and stunting, underweight, and wasting in children are inversely related. Despite the efforts by multiple organizations at the global level, the prevalence of obesity continues to increase incessantly [11]. India ranks 3^rd^ in the Global Obesity Index. It is estimated that approximately 135 million Indians are affected by obesity, which differs due to age, gender, geographic environment, genetics, socioeconomic status, lifestyle, *etc*. As per the 2015 ICMR-INDIAN report, central obesity and its prevalence rate varied from 16.9-36.3% and 11.8-31.3% respectively. Studies have indicated that women are at higher risk of being affected by obesity. Obesity has thus been identified as a medical and financial burden. The World Health Organisation (WHO) estimates that roughly 1.9 billion adults globally (nearly 39% of the world's population) are overweight. More than 650 million of these people (about 13% of the global population) are obese [12, 13]. Abdominal obesity significantly contributes to CVD. Obesity prevalence trends in the United States and across the globe demonstrate the tremendous influence that obesity will continue to have on Cardiovascular incidence and prevalence worldwide. CVD is expected to cause 17.9 million deaths per year. The Global Burden of Disease study estimate of an age-standardized CVD death rate of 272 per 100,000 in India is higher than the global average of 235 per 100,000. The primary cause of disquiet in CVD is its onset at an early age, progression at high speed, and elevated fatality rate. A study comparatively analyzing the statistics of 1990 against 2016 in the context of total death and total disability-adjusted life years (DALYs) reported an elevation of 15.2% *v/s* 28.1% and 6.9% *v/s* 14.1%, respectively. However, with adequate physical activity, proper diet, and awareness, it could be preventable. Desperate efforts are needed to develop a comprehensive understanding of obesity emerging in endemic proportions to manage it [14-16, 153] effectively.

### 6.3 Avoiding Tobacco and Limiting Alcohol Consumption

Avoiding tobacco use and limiting alcohol consumption reduces the risk of obesity and cardiovascular diseases through various mechanisms. Smoking damages the inner lining of blood vessels, leading to endothelial dysfunction. By avoiding tobacco use, endothelial function can improve, promoting better blood flow and reducing the risk of atherosclerosis, heart attacks, and strokes [154]. Tobacco smoke contains numerous harmful chemicals that promote inflammation and oxidative stress in the body. By quitting smoking, inflammation levels decrease, reducing the risk of chronic inflammatory conditions associated with obesity and cardiovascular diseases. Smoking raises blood pressure by causing vasoconstriction and increasing heart rate. By avoiding tobacco use, blood pressure can return to normal levels, reducing the strain on the cardiovascular system and lowering the risk of hypertension-related complications [155]. Alcohol is calorie-dense and can contribute to weight gain and obesity. By limiting alcohol consumption, individuals can reduce excess calorie intake and better manage their weight, decreasing the risk of obesity-related cardiovascular diseases [156] NIAAA, 2020). Excessive alcohol consumption can elevate blood pressure levels. By reducing alcohol intake, blood pressure can be better regulated, reducing the risk of hypertension and its associated cardiovascular complications. Moderate alcohol consumption, defined as one drink per day for women and up to two drinks per day for men, has been associated with a reduced risk of cardiovascular diseases. However, excessive alcohol consumption can negate these benefits. Limiting alcohol intake to moderate levels can provide a potential cardioprotective effect. By avoiding tobacco use and limiting alcohol consumption, individuals can mitigate the adverse impact on cardiovascular health, reduce the risk of obesity, and improve overall well-being [157, 158].

### 6.4 Stress Management

Stress management plays a significant role in reducing the risk of obesity and cardiovascular diseases through the following mechanisms: Chronic stress triggers the release of stress hormones such as cortisol, which can increase appetite and promote the accumulation of abdominal fat. By effectively managing stress, the body's hormonal balance can be regulated, reducing the likelihood of weight gain and obesity. Engaging in stress-reducing activities such as exercise, meditation, deep breathing, and practicing relaxation techniques can help individuals avoid unhealthy coping mechanisms like emotional eating or excessive alcohol consumption [159-161]. By adopting healthier strategies to manage stress, individuals can prevent weight gain and reduce the risk of cardiovascular diseases associated with unhealthy behaviors. Effective stress management techniques, such as relaxation exercises or engaging in enjoyable activities, can help lower blood pressure, reducing the risk of hypertension and related cardiovascular complications. It also reduces inflammation markers in the body, promoting better overall health. Chronic stress can interfere with sleep quality and duration, which has been associated with an increased risk of obesity and cardiovascular diseases [8, 9]. By managing stress, individuals can promote better sleep hygiene, which in turn supports overall health and reduces the risk of weight gain and cardiovascular complications. Stress management is often linked to adopting healthier lifestyle behaviors. When individuals effectively manage stress, they are more likely to engage in regular physical activity, make healthier food choices, maintain a balanced diet, and get adequate sleep. These behaviors collectively contribute to weight management and reduce the risk of obesity and cardiovascular diseases. By implementing stress management techniques, individuals can positively impact their physical and mental well-being, reducing the risk of obesity and cardiovascular diseases. It is important to find strategies that work best for each individual and seek professional guidance if needed [7].

## CONCLUSION

In summary, the review underscores the urgent global need to address the intertwined challenges of obesity, cardiovascular health, and post-COVID complications. Obesity poses significant risks to cardiovascular health, contributing to conditions such as coronary artery disease, heart failure, hypertension, and stroke through mechanisms like chronic inflammation and insulin resistance. The post-COVID-19 health landscape has added complexity to global health, with survivors and the general populace facing potential cardiovascular complications, particularly when coupled with obesity. To mitigate long-term consequences, comprehensive strategies are vital, focusing on lifestyle management involving dietary changes, physical activity, behavioural therapies, and patient education. Prioritizing these lifestyle modifications over medical interventions is crucial, especially for individuals with severe obesity or uncontrolled risk factors. Innovative research areas, including gut microbiota regulation and novel drug development, offer promising avenues. Collaboration between healthcare practitioners, researchers, and policymakers is essential to recognize underlying processes, identify high-risk populations, and implement effective preventive methods, ultimately fostering a healthier future for all.

## Figures and Tables

**Fig. (3):**
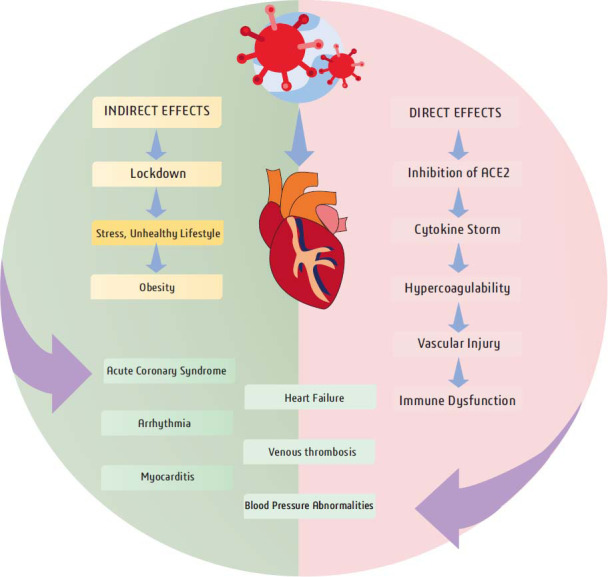
Post-COVID cardiovascular sequelae: Unraveling the mechanisms of action.

## LIST OF ABBREVIATIONS

WHO: World Health Organisation
CDC: Centre Disease Control
CVDs: Cardiovascular Illnesses
ASCVD: Atherosclerosis and Cardiovascular Illnesses
SHS: Second Hand Smoking
IR: Insulin Receptor
ATMs: Adipose Tissue Macrophages
VLDL: Very Low-Density Lipoproteins
REM: Rapid Eye Movement
OSAS: Obstructive Sleep Apnea Syndrome

## DECLARATION

During the preparation of this work, the author(s) used ChatGPT, an AI language model provided by OpenAI, to enhance the English language of the manuscript. The author(s) reviewed, edited, and revised the output to align with their intended meaning and take full responsibility for the content of this publication.
